# A Hybrid Harmonic Curve Model for Multi-Streamer Hydrophone Positioning in Seismic Exploration

**DOI:** 10.3390/s24248025

**Published:** 2024-12-16

**Authors:** Kaiwei Sang, Cuilin Kuang, Lingsheng Lv, Heng Liu, Haonan Zhang, Yijun Yang, Baocai Yang

**Affiliations:** 1 School of Geosciences and Info-Physics, Central South University, Changsha 410083, China; sangkaiwei@csu.edu.cn (K.S.); lingshenglv@csu.edu.cn (L.L.); cusliuheng@csu.edu.cn (H.L.); zhanghn7@cosl.com.cn (H.Z.); yyj123_y@csu.edu.cn (Y.Y.); 2 Geophysical Division of China Oilfield Services Ltd., Tianjin 300451, China; ybc121@csu.edu.cn; 3 National Engineering Research Center of Offshore Oil and Gas Exploration, Beijing 100028, China

**Keywords:** towed streamer seismic exploration, hydrophone positioning, hybrid harmonic function model, large curvature curve fitting, polynomial model

## Abstract

Towed streamer positioning is a vital and essential stage in marine seismic exploration, and accurate hydrophone coordinates exert a direct and significant influence on the quality and reliability of seismic imaging. Current methods predominantly rely on analytical polynomial models for towed streamer positioning; however, these models often produce significant errors when fitting to streamers with high curvature, particularly during turning scenarios. To address this limitation, this study introduces a novel multi-streamer analytical positioning method that uses a hybrid harmonic function to model the three-dimensional coordinates of streamers. This approach mitigates the substantial modeling errors associated with polynomial models in high-curvature conditions and better captures the dynamic characteristics of streamer fluctuations. Firstly, the mathematical model for the hybrid harmonic function is constructed. Then, the algorithmic implementation of the model is detailed, along with the derivation of the error equation and the multi-sensor fusion solution process. Finally, the validity of the model is verified using both simulated and field data. The results demonstrate that, in the turning scenario without added error, the proposed harmonic model improves simulation accuracy by 35.5% compared to the analytical polynomial model, and by 27.2% when error is introduced. For field data, accuracy improves by 18.1%, underscoring the model’s effectiveness in significantly reducing errors associated with polynomial models in turning scenarios. The performance of the harmonic function model is generally comparable to that of the polynomial model in straight scenarios.

## 1. Introduction

Towed streamer seismic exploration has become a mainstream technology in offshore oil and gas exploration due to its high dynamic capacity and operational efficiency. This process involves an exploration vessel towing seismic gun arrays, streamers, and other equipment along a predetermined survey line. At designated shot points, seismic waves from gun arrays are generated and propagate through the seawater until they encounter subsea layers, resulting in reflections and refractions. These seismic signals are then captured by hydrophones attached to the streamers. By analyzing the recorded data, the researchers can preliminarily detect the seabed geological structure and provide reference for subsequent oil and gas exploration. In this context, the precise coordinate of hydrophones not only directly influences seismic quality but also affects the stratigraphy interpretation, thereby reducing the success rate of exploration [1,2]. However, achieving accurate positioning is challenging in practice, given the variable environmental conditions, dynamic operating circumstances, and the complexity of large-scale towing systems, often comprising a dozen streamers, each ranging from several to dozens of kilometers in length. Consequently, ensuring accurate hydrophone positioning has become a critical issue in marine exploration.

The development of streamer positioning methods has progressed through three primary stages: single-streamer positioning, multi-streamer numerical positioning, and multi-streamer analytical positioning. In early offshore exploration, a single streamer equipped only with a magnetic sensor was used. In the 1980s, Gilbert proposed a single-streamer analytical model that used polynomial functions to establish relationships with observations such as azimuth, depth, and acoustic distance, thereby describing the streamer. The polynomial coefficients were estimated using maximum likelihood, enabling the determination of the streamer shape and hydrophone position based on the design configuration [3,4]. Similarly, a single streamer can also be modeled using curve integrals, arc segments, or piecewise polynomials based on azimuth, distance, or known points [5,6,7]. In general, by employing magnetic compass data to fit model parameters, they directly or indirectly determine the morphology and coordinates of the streamer, yielding hydrophone coordinates, collectively referred to as analytical methods. Analytical methods offer advantages such as a rigorous mathematical foundation, a minimal number of parameters, an intuitive computational process, and a smooth curve consistent with the geometric characteristics [3,4,5,6,7,8,9,10]. However, early analytical methods mainly focus on single-streamer positioning and do not fully account for the complexities of multi-streamer operations.

Since the 1990s, seismic exploration technology has evolved from 2D (two-dimensional) to 3D and 4D, driven by the growing demand for high-resolution seismic data. Multi-streamer configurations, now standard in the industry, are equipped with GNSS (Global Navigation Satellite System) buoys and acoustic networks to enhance positioning accuracy [11,12,13,14,15,16,17]. Numerical methods obtain node coordinates through coordinate integration and acoustic network adjustments, determining hydrophone coordinates based on these node coordinates and their relational configurations. These methods typically model the streamer as a series of connected points along polylines or arcs, using azimuth and distance measurements between streamer points for network adjustments. Several variants of these numerical methods have been developed, such as quadratic fitting, which is applied after acoustic network adjustment, and coordinates are determined using a Kalman filter combined with interpolation to enhance network stability [18,19,20,21,22]. Yi et al. refined coordinate recursions and evaluated positioning accuracy, confirming that components such as arcs, polynomial curves, and integral polylines meet the requirements of seismic exploration. Yi also integrated virtual distance observations from compasses into the acoustic network, enhancing network connectivity and positioning stability [20,21,22,23]. Gikas provided a detailed discussion of modeling approaches, including physical and empirical numerical methods. Various curve-fitting methods such as polyline, circular arc, cubic spline, polynomial, and harmonic fitting were compared, ultimately selecting polynomial fitting for its ability to represent the entire shape with continuity and its compatibility with the Kalman filter [12]. These multi-streamer positioning approaches offer adaptability, flexibility, and ease of implementation. However, existing approaches struggle to directly link observations to hydrophone coordinates and couple multiple data loosely. This lack of deep data fusion diminishes the utility of azimuth and distance data, reducing the robustness of the positioning model and its accuracy assessments. Furthermore, using discrete nodes or arcs to approximate a smooth curve presents trade-offs: increasing the number of points improves shape accuracy but also adds more unknown parameters, such as node coordinates and computation time, while reducing points speeds up computation but compromises the representation of the streamer’s inherent geometric smoothness.

**Figure 1:**
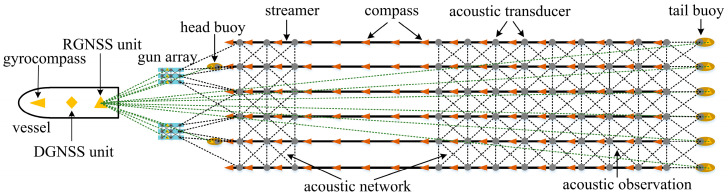
Towed streamer exploration and positioning network.

Currently, seismic exploration using multi-streamers with multiple seismic sources has become standard practice. As the number of sensors on streamers increases, so does the demand for precise hydrophone positioning. The positioning method has evolved from numerical to analytical approaches for multi-streamers. The analytical methods establish functional relationships between observations and shape, determining hydrophone coordinates through morphological parameters. For instance, Yu et al. proposed a multi-streamer analytical positioning method based on polynomial curve fitting, extending the analytical approach to multi-streamers. This method uses magnetic compass bearings, acoustic distance, and depth observations from all streamers to estimate parameters collectively. Tests with field data validated its effectiveness and practicality, grouping and refining both homologous and heterogeneous data through variance component estimation, resulting in more accurate hydrophone coordinates [24,25]. However, fitting a streamer during turning increases modeling errors, particularly in sections with fewer acoustic observations, such as those in the front-rear acoustic network configurations shown in Figure 1. Duan et al. introduced a rigorous positioning model based on curve integration, leveraging the differential relationships of any point on the streamer to determine its shape and coordinates. Their model demonstrated higher accuracy than numerical methods in both simulations and field data. They also suggested using cubic spline curves to fit the streamer with continuity constraints, which improves accuracy and enforces and effectively reduces modeling errors in complex turning scenarios [26,27]. Compared to numerical methods, multi-streamer analytical positioning offers distinct advantages. In terms of parameter solving and practical application, the analytical model directly represents observations along the streamer as a function of the base curve using methods such as polynomials and cubic spline, significantly reducing unknown parameters. As the number of nodes on the streamer increases, the advantages of minimizing unknowns become even more apparent, enhancing solution efficiency and drastically reducing complexity. This capability meets the demands of large-scale, high-complexity, multi-sensor integration in time-sensitive streamer navigation and positioning applications. In terms of model simplification and stability, the analytical model can directly calculate the coordinates of any point on the streamer without additional observations of that point or readjustment. By substituting the offset of the point into the function, coordinates can be quickly obtained, greatly simplifying the solution process and making it well-suited for scenarios requiring a high number of rapid observations, such as a dozen streamers with a full network configuration.

Towed streamers inevitably require steering, rudder adjustments, and turnaround during large area explorations. While seismic data acquired under streamer bending conditions are generally considered substandard, accurately modeling the shape remains essential. In turning scenarios, complex currents and environmental factors often cause sensor observation outliers and streamer shape anomalies, such as knots and crossings. Accurate modeling of large-curvature streamers is critical to minimizing these irregularities and ensuring operational efficiency for subsequent survey lines [27,28]. Operationally, large curvature is defined by the relationship between the streamer length and the steering radius. If the steering radius is smaller than the radius of a semicircle formed by the length of the streamer, the curvature is considered large. However, existing polynomial models often result in significant modeling errors when applied to large curvature sections, making it difficult to accurately represent complex shapes in turning scenarios. Additionally, high-order polynomials are susceptible to the Runge’s phenomenon, which can cause substantial deviations between fitted curves and actual shapes [28]. While cubic spline curves can effectively reduce modeling errors in high-curvature conditions, streamers with insufficient acoustic observations may exhibit ill-posed adjustments that can distort positioning results. Although the rigorous curve integral model provides higher accuracy in streamer representation, it requires integral calculations, which increase computational time. Therefore, this paper proposes a novel multi-streamer analytical positioning model based on hybrid harmonic function, which addresses the significant modeling errors of polynomial models in high-curvature scenarios while preserving computational efficiency and enhancing the accuracy and stability of hydrophone positioning.

## 2. Methodology

### 2.1. Hybrid Harmonic Model

Multi-streamer positioning is a form of multi-sensor fusion positioning that relies on various types of observations to construct a reliable and rigorous positioning model. In seismic exploration, the sensors on the vessel include DGNSS (Differential GNSS) and gyrocompass. DGNSS is responsible for determining the three-dimensional absolute coordinates of the vessel within the geocentric geoid coordinate system, generally a satellite augmentation system, while the gyrocompass provides the true azimuth [29,30]. The vessel tows gun arrays and streamers, which are equipped with RGNSS (Relative GNSS) buoys at both the head and tail to measure baseline vectors relative to the reference station on the vessel. Both the gun arrays and streamers are located underwater and are outfitted with compass sensors, acoustic sensors, depth sensors, and sound velocity sensors, which are used to measure various physical observations, including magnetic azimuth, acoustic propagation time, depth, and sound velocity, respectively, as illustrated in Figure 1.

If the offset of any point on the streamer is denoted as s and the coordinate vector as $x={\left[n,e,u\right]}^{T}$, the analytical positioning model of the streamer can be expressed as Equation (1). By analyzing the positioning observations, the parameters of the analytical model can be estimated, allowing the coordinates of the streamer at any point to be derived directly or indirectly through the model function.

(1) $$x=f\left(s,p\right)=\left[\begin{array}{c} f_{n}\left(s,p_{n}\right)\\ f_{e}\left(s,p_{e}\right)\\ f_{u}\left(s,p_{u}\right) \end{array}\right]$$

This equation, p represents the model parameter to be estimated in the function f, while $f_{n}(s,p_{n})$/$f_{e}(s,p_{e})$/$f_{u}(s,p_{u})$ is the expression of the model function f(s,p) concerning the north/east/up (n/e/u) components. The parameters $p_{n}$/$p_{e}$/$p_{u}$ correspond to the model parameters associated with the n/e/u components, respectively.

**Figure 2:**
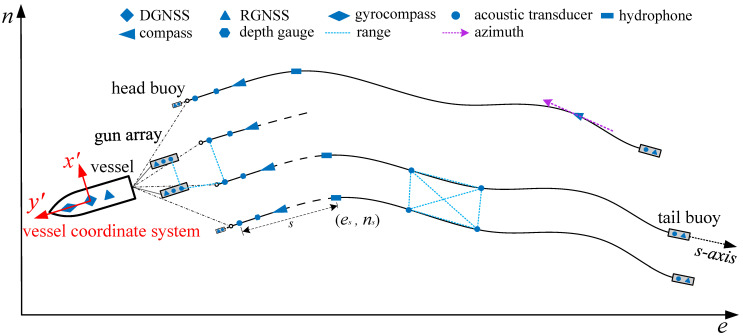
Hybrid harmonic function positioning model.

In actual operations, the streamer typically exhibits the characteristics of a smooth curve. Each streamer experiences similar tension, resulting in an overall curvature that fluctuates due to environmental factors such as currents. Consequently, a harmonic function model that captures these fluctuating characteristics can be employed to model and describe the streamer, enabling more accurate analytical positioning. The hybrid harmonic function is represented by a constant term, a trend term, and a harmonic function term, and the harmonic function term accounts for the fluctuations of the streamer and theoretically reduces modeling errors. The hybrid harmonic function model is established by the functional relationship between the coordinates of any point on the streamer and its offset, as illustrated in Figure 2. Taking a single streamer as an example, the mathematical model fitted by the hybrid harmonic function can be expressed as:

(2) $$f(s)=a_{0}+a_{1}\times s+\overset{N}{\underset{n=2}{\sum }}(a_{n}\times \sin(\frac{(n-1)m\pi }{L}\times s))$$

Among them, the design offset of the point on the streamer is expressed as s, and $a_{0}$ represents the constant term that describes the overall shift of the coordinate components; $a_{1}\times s$ is the trend term, indicating the trend changes of the coordinate components; and $\sum_{n=2}^{N}(a_{n}\times \sin(\frac{(n-1)m\pi }{L}\times s))$ denotes the harmonic function term, which describes the periodic fluctuation characteristics of the coordinate components. Here, $a_{n}$ indicates the harmonic function coefficients, while m represent the trigonometric periodic components of the harmonic function term during the fitting process. *N* refers to the order of the hybrid harmonic function, and L is the total length of the streamer. To account for stretching and avoid oscillatory fitting at the ends, L is typically set slightly greater than the nominal length.

Knowing the offset values of each point on the streamer and projecting the hybrid harmonic function curve to different coordinate components, the coordinate components of any point $\left(x_{i},y_{i},z_{i}\right)$ on the streamer can be expressed as:

(3) $$\left\{\begin{array}{c} x_{i}=f_{x}\left(s_{i}\right)=a_{0}+a_{1}\times s_{i}+\overset{N}{\underset{n=2}{\sum }}(a_{n}\times \sin(\frac{(n-1)m\pi }{L}\times s_{i}))=S_{i}^{T}A\\ \begin{array}{c} y_{i}=f_{y}\left(s_{i}\right)=b_{0}+b_{1}\times s_{i}+\overset{N}{\underset{n=2}{\sum }}(b_{n}\times \sin(\frac{(n-1)m\pi }{L}\times s_{i}))=S_{i}^{T}B\\ z_{i}=f_{z}\left(s_{i}\right)=c_{0}+c_{1}\times s_{i}+\overset{N}{\underset{n=2}{\sum }}(c_{n}\times \sin(\frac{(n-1)m\pi }{L}\times s_{i}))=S_{i}^{T}C \end{array} \end{array}\right.$$

This equation $\left(x_{i},y_{i},z_{i}\right)$ denotes the coordinate component of the point i on the streamer in different dimensions, while $f_{x}\left(s_{i}\right)/f_{y}\left(s_{i}\right)/f_{z}\left(s_{i}\right)$ represents the hybrid harmonic function relationship between the offset $s_{i}$ of the point i and the coordinates in different dimensions. Here, $S_{i}={\left[s_{i}^{0},s_{i}^{1},\sin(\frac{1m\pi }{L}\times s_{i}),\ldots .,\sin(\frac{(n-1)m\pi }{L}\times s_{i})\right]}^{T}$ is the design offset matrix of the point i along the streamer direction. m is usually taken as 1. The vector of coefficients corresponding to the coordinate component is denoted as A/B/C, denoted as $A={\left[a_{0},a_{1},\cdots ,a_{N}\right]}^{T}$, $B={\left[b_{0},b_{1},\cdots ,b_{N}\right]}^{T}$, and $C={\left[c_{0},c_{1},\cdots ,c_{N}\right]}^{T}$, respectively. N is typically taken as 5 to 7.

The hybrid harmonic function model is a multi-streamer analytical positioning method that enables the determination of the coordinates of any point on the streamer while maintaining stable computational efficiency. It effectively reflects the fluctuating geometric characteristics of the streamer, and the fitting process streamer determines the position of the hydrophone. Furthermore, the harmonic function terms in the model are homogeneous terms that can effectively mitigate the oscillation issues, referred to as the Runge’s phenomenon, that can occur at the edges of the fitting interval.

### 2.2. Multi-Streamer Positioning Method

#### 2.2.1. Observation Equations and Linearization

To solve for the coefficients of the hybrid harmonic function model, it is necessary to establish a functional relationship among the coordinate observations, compass bearings observations, acoustic distance observations, depth sensor observations, and the coefficients to be estimated. The specific derivations of the observation equations are provided below:

(1) **Coordinates**: Coordinate observations are typically high-accuracy coordinates, such as RGNSS buoy nodes or predicted virtual nodes:

(4) $$r_{i}=\left[\begin{array}{c} n_{s}\\ e_{s}\\ u_{s} \end{array}\right]=f(s_{i})=\underset{H_{f_{s}}}{\underset{︸}{{\left[\begin{array}{ccc} S_{i} & & \\ & S_{i} & \\ & & S_{i} \end{array}\right]}_{3\times 3(N+1)}}}\underset{X}{\underset{︸}{{\left[\begin{array}{c} A\\ B\\ C \end{array}\right]}_{3(N+1)\times 1}}}$$

where $r_{i}$ is the coordinate vector of the point i, $S_{i}$ is the design offset matrix of the point i on the streamer, and N is the order of the hybrid harmonic function.

Linearization of the above equation using approximate parameters $\left(A^{0},B^{0},C^{0}\right)$:

(5) $$f(s_{i})=\left[\begin{array}{c} n_{s}\\ e_{s}\\ u_{s} \end{array}\right]=H_{f_{s}}\left[\begin{array}{c} \delta A\\ \delta B\\ \delta C \end{array}\right]+\left[\begin{array}{c} n_{s}^{0}\\ e_{s}^{0}\\ u_{s}^{0} \end{array}\right]+\left[\begin{array}{c} \varepsilon_{n_{s}}\\ \varepsilon_{e_{s}}\\ \varepsilon_{u_{s}} \end{array}\right]$$

where δA/δB/δC represents the corrections to the coefficients corresponding to different coordinate dimensions; $\left[\begin{array}{ccc} n_{s}^{0} & e_{s}^{0} & u_{s}^{0} \end{array}\right]$ is the approximate value of the coordinate vector at the curve s; and $\left[\begin{array}{ccc} \varepsilon_{n_{s}} & \varepsilon_{e_{s}} & \varepsilon_{u_{s}} \end{array}\right]$ is the error in the observations.

(2) **Acoustic distance:** Acoustic distance observations are the most numerous in streamer positioning. For generality, assuming they are cross-line observations, the observation equation is:

(6) $$r_{H_{i}K_{j}}={\left\|x_{H_{i}}-x_{K_{j}}\right\|}_{2}={\left\|f\left(s_{H_{i}}\right)-f\left(s_{K_{j}}\right)\right\|}_{2}={\left({\left[\begin{array}{c} S_{H_{i}}A_{H}-S_{K_{j}}A_{K}\\ S_{H_{i}}B_{H}-S_{K_{j}}B_{K}\\ S_{H_{i}}B_{H}-S_{K_{j}}B_{K} \end{array}\right]}^{T}\left[\begin{array}{c} S_{H_{i}}A_{H}-S_{K_{j}}A_{K}\\ S_{H_{i}}B_{H}-S_{K_{j}}B_{K}\\ S_{H_{i}}B_{H}-S_{K_{j}}B_{K} \end{array}\right]\right)}^{1/2}+\varepsilon_{r_{H_{i}K_{j}}}$$

where $r_{H_{i}K_{j}}$ represents the distance observations and $x_{H_{i}}$ and $x_{K_{j}}$ are the acoustic transducer coordinates of point i on streamer H and point j on streamer K, respectively. $S_{H_{i}}$ and $S_{K_{j}}$ are the offset of the acoustic pair on the streamer, and $\varepsilon_{r_{H_{i}K_{j}}}$ is the observed error.

Linearization of the observation equation yields:

(7) $$r_{H_{i}K_{j}}=\frac{1}{r_{H_{i}K_{j}}^{0}}{\left[\begin{array}{c} \begin{array}{c} \left(S_{H_{i}}A_{H}^{0}-S_{K_{j}}A_{K}^{0}\right)S_{H_{i}}\\ \left(S_{H_{i}}B_{H}^{0}-S_{K_{j}}B_{K}^{0}\right)S_{H_{i}} \end{array}\\ \begin{array}{c} \left(S_{H_{i}}C_{H}^{0}-S_{K_{j}}C_{K}^{0}\right)S_{H_{i}}\\ \left(S_{K_{j}}A_{K}^{0}-S_{H_{i}}A_{H}^{0}\right)S_{K_{j}} \end{array}\\ \begin{array}{c} \left(S_{K_{j}}B_{K}^{0}-S_{H_{i}}B_{H}^{0}\right)S_{K_{j}}\\ \left(S_{K_{j}}C_{K}^{0}-S_{H_{i}}C_{H}^{0}\right)S_{K_{j}} \end{array} \end{array}\right]}^{T}\left[\begin{array}{c} \begin{array}{c} \delta A_{H}\\ \delta B_{H} \end{array}\\ \begin{array}{c} \delta C_{H}\\ \delta A_{K} \end{array}\\ \begin{array}{c} \delta B_{K}\\ \delta C_{K} \end{array} \end{array}\right]+r_{H_{i}K_{j}}^{0}+\varepsilon_{r_{H_{i}K_{j}}}$$

where $\delta A_{H}/\delta B_{H}/\delta C_{H}$ and $\delta A_{K}/\delta B_{K}/\delta C_{K}$ are the corrections to the coefficients of the hybrid harmonic function corresponding to the different dimensional coordinates of point i on streamer H and point j on streamer K; $r_{H_{i}K_{j}}^{0}$ is an approximation of the distance observations; and $\varepsilon_{r_{H_{i}K_{j}}}$ is the observation error.

The in-line observations equation represents a special case of cross-line, which can be simplified as:

(8) $$r_{ij}=\frac{1}{r_{ij}^{0}}{\left[\begin{array}{c} \left(S_{i}-S_{j}\right)A^{0}\left(S_{i}-S_{j}\right)\\ \left(S_{i}-S_{j}\right)B^{0}\left(S_{i}-S_{j}\right)\\ \left(S_{i}-S_{j}\right)C^{0}\left(S_{i}-S_{j}\right) \end{array}\right]}^{T}\left[\begin{array}{c} \delta A\\ \delta B\\ \delta C \end{array}\right]+r_{ij}^{0}+\varepsilon_{r_{ij}}$$

(3) **Azimuth**: The observation equation for azimuth observations is:

(9) $$\tan\alpha =\frac{\partial n}{\partial e}=\frac{\partial f_{n}\left(s\right)}{\partial f_{e}\left(s\right)}=\frac{\frac{\partial f_{n}\left(s\right)}{\partial s}}{\frac{\partial f_{e}\left(s\right)}{\partial s}}=\frac{f_{n}^{\prime }\left(s\right)}{f_{e}^{\prime }\left(s\right)}$$

The azimuth $\alpha_{s}$ at streamer s can be expressed as:

(10) $$\alpha_{s}=\arctan_{2}\left(\frac{D_{s}B}{D_{s}A}\right)+\varepsilon_{\alpha_{s}}$$

(11) $$D_{s}=\frac{\partial S}{\partial s}=\left[0,s^{0},\frac{1mn\pi }{L}\cos(\frac{1mn\pi s}{L}),\frac{2mn\pi }{L}\cos(\frac{2mn\pi s}{L}),\cdots ,\frac{(N-1)mn\pi s}{L}\cos(\frac{(N-1)mn\pi s}{L})\right]$$

where $\varepsilon_{\alpha_{s}}$ denotes the error in the azimuthal observations. Observations are typically corrected for magnetic declination based on the World Magnetic Model (WMM). The partial differential equation based on the inverse tangent function can be expressed as:

(12) $$\partial (\arctan_{2}(\frac{n}{e}))=\frac{-n}{e^{2}+n^{2}}\partial e+\frac{e}{e^{2}+n^{2}}\partial n$$

Linearization of the above equations using approximation coefficients $A^{0}/B^{0}$ yields:

(13) $$\alpha_{s}=\frac{1}{D_{s}\left(A^{0}A^{0T}+B^{0}B^{0T}\right){D_{s}}^{T}}{\left[\begin{array}{c} -D_{s}B^{0}D_{s}\\ D_{s}A^{0}D_{s} \end{array}\right]}^{T}\left[\begin{array}{c} \delta A\\ \delta B \end{array}\right]+\alpha_{s}^{0}+\varepsilon_{\alpha_{s}}$$

(4) **Depth**: Depth observations are obtained by depth sensors on the streamer, where depth is usually positive downwards. The depth observation equation can be expressed as follows:

(14) $$h_{s}=-SC+\varepsilon_{h_{s}}$$

Linearization of the above equation using approximate parameters $C^{0}$ results in:

(15) $$h_{s}=-S\delta C-h_{s}^{0}+\varepsilon_{h_{s}}$$

#### 2.2.2. Parameter Estimation and Hydrophone Position Calculation

The model calculation process can be divided into data preprocessing and adjustment calculations. Data preprocessing includes detecting outliers, using spline interpolation to derive the azimuth of any point along each streamer, and employing curve integration to obtain the approximate coordinates of the acoustic and compass nodes [31]. Subsequently, the coordinates of nodes are then solved using curve integration to derive the initial harmonic function coefficients. Finally, the curve coefficients are obtained through adjustment calculations. Using the observation Equations (4)–(15) for various types of positioning observations, generalized least squares fusion can be employed to estimate these equations, thus determining the parameters for each streamer. Virtual observations can be effectively used by generalized least squares to enhance the stability of solutions [32], integrating the virtual coordinates of acoustic and compass nodes as well as hydrophones within the parameter solution equations.

(16) $$\underset{Y}{\underset{︸}{\left[\begin{array}{c} \begin{array}{c} Y_{1}\\ Y_{2} \end{array}\\ \begin{array}{c} \vdots \\ Y_{n} \end{array} \end{array}\right]}}=\underset{H}{\underset{︸}{\left[\begin{array}{c} H_{1}\\ H_{2}\\ \vdots \\ H_{n} \end{array}\right]}}\delta X+\underset{Y^{0}}{\underset{︸}{\left[\begin{array}{c} \begin{array}{c} Y_{1}^{0}\\ Y_{2}^{0} \end{array}\\ \begin{array}{c} \vdots \\ Y_{n}^{0} \end{array} \end{array}\right]}}+\underset{\xi }{\underset{︸}{\left[\begin{array}{c} \xi_{1}\\ \xi_{2}\\ \vdots \\ \xi_{n} \end{array}\right]}}$$

where Y is the vector of observations, H is the design matrix, $Y^{0}$ is the initial value of observations calculated based on the approximate parameter $X^{0}$, and ξ is the observation error vector. The stochastic model is constructed using the a priori accuracy of the observations, and refine weighting schemes can be employed using variance component estimation [25].

(17) $$E(\xi )=0,\Sigma_{\xi }=\left[\begin{array}{cccc} \sigma_{01}^{2}p_{1}^{-1} & & & \\ & \sigma_{02}^{2}p_{2}^{-1} & & \\ & & \ddots & \\ & & & \sigma_{0n}^{2}p_{n}^{-1} \end{array}\right]$$

Finally, the parameters $\hat{X}=[\begin{array}{ccc} {\hat{A}}^{T} & {\hat{B}}^{T} & {\hat{C}}^{T} \end{array}]$ of the harmonic function are obtained. Through the function model in Equation (4), the position information of any point on the streamer can be acquired, because the installation positions of the sensors on the streamer are known and usually fixed. At the same time, the covariance of the coordinates of any point on the streamer can be derived according to the law of error propagation.

(18) $$\hat{f_{s}}=H_{f_{s}}\hat{X}$$

(19) $$\Sigma_{\hat{f_{s}}}=H_{f_{s}}\Sigma_{\hat{X}}H_{f_{s}}^{T}$$

where $\hat{f_{s}}$ is the position on the streamer at the offset s, and $\Sigma_{\hat{f_{s}}}$ is the variance–covariance matrix at the position.

## 3. Experiments and Result Discussion

This comparative analysis was conducted between the polynomial model and the hybrid harmonic model using simulated and field data to evaluate the accuracy and stability and validate the practical application.

### 3.1. Simulation Test

**Figure 3:**
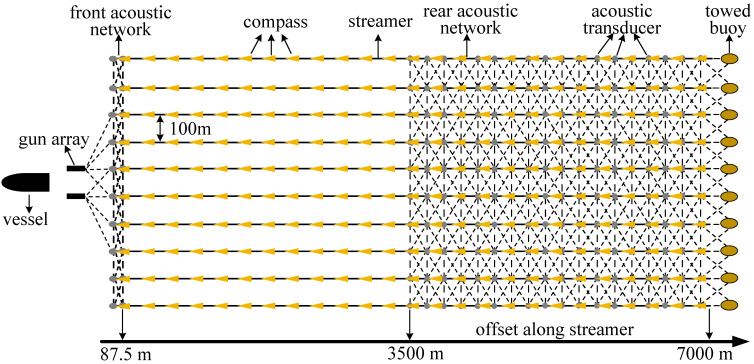
Simulated positioning network configuration.

The simulation data were generated by SimStr 1.0, software independently developed by the author’s research team as a simulation platform based on the dynamics of streamers. It can simulate various types of observations and produce outputs in the standard marine seismic exploration positioning data exchange formats, P2/94 and P1/90, thereby effectively meeting the evaluation needs of the positioning models [33]. In the simulation, the survey vessel was equipped with DGNSS sensors and a gyrocompass, while the gun arrays and tail buoys were equipped with RGNSS sensors. A total of 10 streamers, each 7000 m long and spaced 100 m apart, were towed. Each streamer was equipped with 25 compasses spaced 300 m apart, with an additional compass mounted at the tail. The tail buoys had acoustic transducers, as shown in Figure 3, for the simulated data positioning network configuration. The acoustic network employed the commonly used front-rear configurations, with two acoustic transducers installed at the head of each streamer spaced 75 m apart, forming the front acoustic network with the transducers on the gun array. In the middle and rear sections (offsets from 3500 m to 7000 m), 18 acoustic transducers were installed at 200 m intervals, forming the rear acoustic network with the tail buoy acoustic transducers. Figure 3 illustrates the configuration of the simulated network. Each streamer was equipped with 561 hydrophones, with an installation interval of 12.5 m. The azimuth of the survey line was set to 0°. Detailed parameters are listed in Table 1.

**Figure 4:**
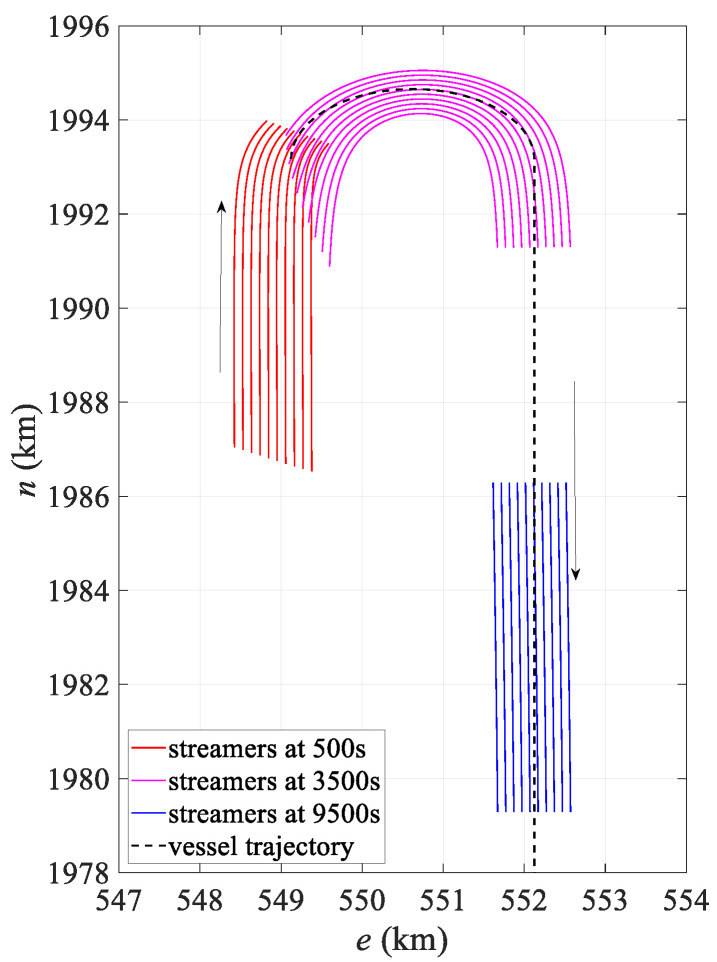
Simulated data trajectory.

The simulation experiment simulated a total of 1000 observation epochs, outputting observations and corresponding true coordinates of the hydrophones every 10 s, which were recorded in the P2/94 and P1/90 files over a total duration of 10,000 s. To test the accuracy and applicability of the hydrophone positioning model under different environmental conditions, two scenarios were simulated: straight and turning. The first 600 epochs represented the turning scenario, while the last 400 epochs represented straight movement. For simulating a large curvature turning scenario, the turning radius was set to 1500 m, with a turning angle of 180°. Figure 4 depicts the shape of the streamer at different times.

The simulated data were divided into two groups to evaluate the hydrophone positioning model. The first group did not include any errors, allowing for an assessment of the modeling errors. The second group introduced random errors to test the accuracy and stability. In order to adequately test for streamer performance, no errors were added to the datum sensors (DGNSS, gyrocompass, gun array, and tail buoy RGNSS). Errors were only introduced into two types of observations: compass bearing and acoustic distance. In practice, the quality of observations from the front compass is usually higher than from the back compass, and the quality of the in-line is usually higher than the cross-line. Therefore, for the compasses in the front section of the streamer (offset < 3500 m), a normally distributed random error with a standard deviation of 0.5° was added, while rear section compasses (offset > 3500 m) had a 1° standard deviation. Acoustic observations within in-line had a 0.75 ms standard deviation, and those cross-line had a 1.50 ms one.

Two positioning models were employed to test both sets of data. The calculated hydrophone coordinates were compared with the true values from the simulation software output in P1/90, according to Equation (20).

(20) $$d^{i}=\frac{1}{n_{r}}\sum_{j=1}^{n_{r}}{\left[{\left({\hat{n}}_{j}^{i}-{\tilde{n}}_{j}^{i}\right)}^{2}+{\left({\hat{e}}_{j}^{i}-{\tilde{e}}_{j}^{i}\right)}^{2}\right]}^{\frac{1}{2}}$$

where: $d^{i}$ denotes the absolute mean deviation of the hydrophone for the i-th epoch; $n_{r}$ is the total number of hydrophones on the streamers; $\left({\hat{n}}_{j}^{i},{\hat{e}}_{j}^{i}\right)$ represents the projected coordinates of the hydrophone $R_{j}$ for the i-th epoch; and $\left({\tilde{n}}_{j}^{i},{\tilde{e}}_{j}^{i}\right)$ represents the true coordinates of the hydrophone for the i-th observation epoch as recorded in the simulation P1/90 file.

To evaluate the accuracy of the hydrophones under different spatial distributions and to test the stability of the streamer in regions where observations are not uniformly distributed (such as the front-middle sections with only compass observations), the deviation of the hydrophone at its design offset was calculated using Equation (21).

(21) $$d_{j}=\frac{1}{n_{t}}\overset{n_{t}}{\underset{i=1}{\sum }}{\left[{\left({\hat{n}}_{j}^{i}-{\tilde{n}}_{j}^{i}\right)}^{2}+{\left({\hat{e}}_{j}^{i}-{\tilde{e}}_{j}^{i}\right)}^{2}\right]}^{\frac{1}{2}}$$

where $d_{j}$ denotes the mean point deviation of the hydrophones at $s_{j}$ for all streamers; $n_{t}$ is the number of epochs.

#### 3.1.1. Without Observation Errors

The P2/94 data without errors were tested using both positioning models. The deviation of the hydrophone point positions for each epoch was calculated according to Equation (20), and the results are presented in Table 2. In the ideal case without errors, the mean deviation of the hydrophones for the polynomial model was approximately 10 m. In the large curvature scenario with a turning radius of 1500 m, the maximum positioning deviation reached 23.6 m, indicating that the polynomial model generally exhibits lower positioning accuracy. Conversely, the mean deviation of the harmonic model was about 6.8 m, which mitigates approximately 35.5% of the modeling error. The maximum deviation was 11.4 m, far less than 23.6 m, overall performing better than the polynomial model. This was because the turning shape had fluctuation, and the harmonic function could be effectively fitted. Moreover, the standard deviation of the mean positioning deviation across epochs was significantly lower than that of the polynomial model, suggesting that the new model can adapt well to the dynamic changes in the shape. The interquartile range showed notable improvement, reduced by 74.7%, indicating that the harmonic model effectively enhanced the stability of the model while simplifying its complexity. Under the straight scenario, the positioning deviations for both models remained small, approximately 0.3 m, with both models demonstrating similar accuracy and stability in describing the simple streamer shape.

**Figure 5:**
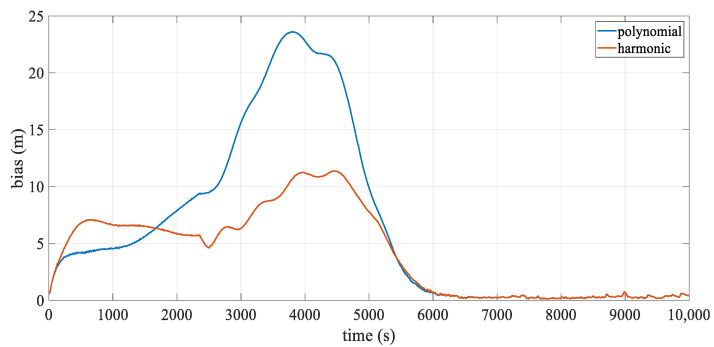
Mean positioning deviation over epoch.

Figure 5 illustrates the mean deviation of the hydrophones over epochs. The accuracy of both analytical models was affected by the change in stream shape, with the polynomial model being particularly sensitive to these variations. Its error increased rapidly, peaking around 3750 s at over 20 m, which corresponded to the streamer’s point of maximum curvature, as shown in Figure 4, especially for the innermost streamer. Although the positioning error of the harmonic model also fluctuated with the shape, it was generally less influenced and remained more stable, effectively mitigating modeling error. After 5500 s, as the streamer shape gradually straightened, the positioning errors for both models decreased sharply. Interestingly, around 500 s, the harmonic model’s error briefly exceeded that of the polynomial model. This slight discrepancy was likely due to only the front section of the streamer being curved at this initial turning phase, causing fluctuations in the harmonic model to propagate to the middle and rear sections, which were not yet significantly bent. However, this effect remained relatively minor overall.

Table 3 shows the statistics of the spatial deviation $d_{j}$ of the streamer, which is the same as $d^{i}$ in the mean. In the turning scenario, the spatial maximum deviation of the polynomial model was about 18.1 m, while the new model deviation was about 11.5 m, which is significantly smaller than that of the polynomial model. Moreover, the interquartile range over spatial deviations was comparable for both models, indicating similar spatial variation.

**Figure 6:**
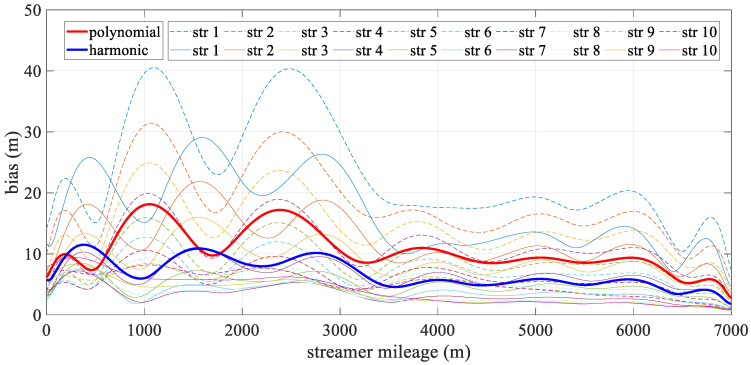
Mean positioning deviation along offset in turning scenario.

**Figure 7:**
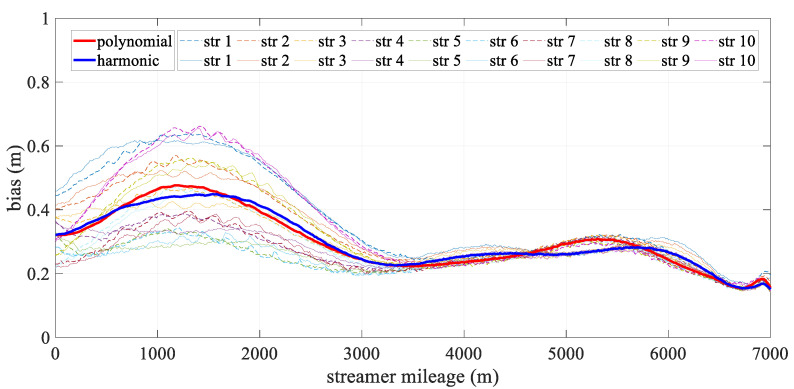
Mean positioning deviation along offset in straight scenario.

Figure 6 depicts the mean deviation along each streamer during the turning scenario. The bold line represents the mean positioning deviation of the hydrophone derived from all streamers for both models, with the new model being particularly effective in reducing errors in the front–middle sections. This concentration of deviation in the front-middle section is due to the fact that, in the front-rear network configurations, only compass observations are used to constrain the modeling, without the benefit of acoustic observations. As a result, errors may accumulate, affecting the overall geometry of the streamer. The harmonic function, however, can effectively estimate the sections that lack acoustic data. The high accuracy at both ends of the streamer was mainly due to the constraints provided by the gun arrays and the tail buoys, which were governed by reference data and the acoustic network. Meanwhile, the accuracy of both analytical models fluctuated. The polynomial model was relatively unstable, primarily due to the uneven distribution of observations along the streamer, while the harmonic function model adapted more effectively to shape changes. As shown in Figure 7, the straight scenario resulted in minor deviations, less than 0.5 m, with both models demonstrating similar levels of accuracy and spatial distribution.

**Figure 8:**
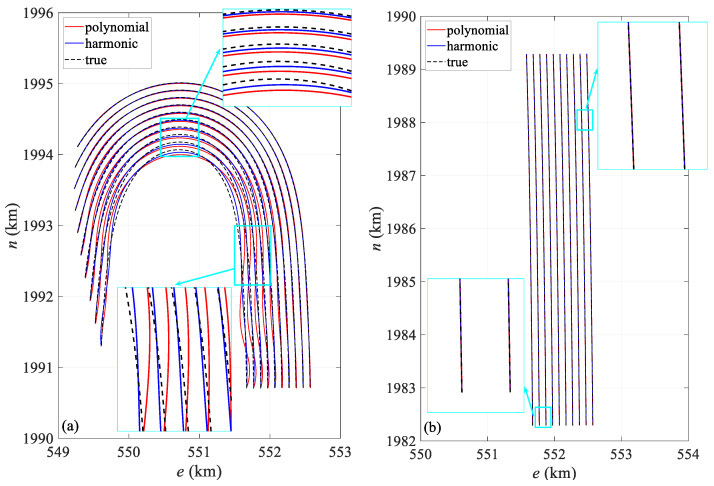
Streamer shape in (**a**) 3790 s turning and (**b**) 8000 s straight scenario.

Figure 8 compares the shapes of the streamer in both the turning and straight scenarios. Figure 8a presents the streamer shape at 3790 s, when the curvature of the streamer is at its maximum, especially the innermost streamer. At this point, the head and tail directions are close to 180°. The polynomial model produces significant distortion, especially in the middle section, causing the positioning results to deviate considerably from the actual position. Both models show slight positional offsets in the middle and tail sections, but the polynomial model exhibits much larger modeling errors, particularly for the innermost streamer. In contrast, the harmonic function model better represents the actual shape, with its calculated shape generally aligning with the true streamer form. The fitted streamer shapes show minor fluctuations compared to the actual shape, primarily due to the discrete node distribution. Higher precision in node placement leads to better conformity with the actual streamer shape. Figure 8b depicts the streamer shape at 8000 s, representing the straight scenario, where both models closely align with the actual shape.

#### 3.1.2. With Observation Errors

Similar to Section 3.1.1, the P2/94 data with errors were tested, and the statistical results are presented in Table 4. It is obvious that in the turning scenario, the polynomial model exhibited a higher error than the harmonic model. While a portion of the error was attributed to modeling inaccuracies, the mean accuracy improved by approximately 27.2%. The interquartile range for the harmonic model was notably smaller, reduced by 74.9%, indicating superior stability and robustness. In the straight scenario, both models showed similar precision. When compared to Table 1, the overall mean positioning deviations for both models in the presence of observation errors increased by 1.63 m and 2.06 m in the turning scenario and by 4.26 m and 4.35 m in the straight scenario, respectively. The increase in deviation was comparable for both models, highlighting their robustness against errors in hydrophone positioning, which is a notable advantage of the analytical method.

**Figure 9:**
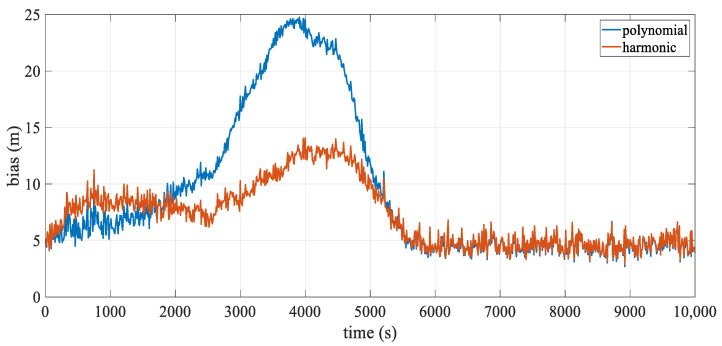
Mean positioning deviation over epoch.

Figure 9 illustrates the mean positioning deviation over epochs. Both models displayed fluctuations due to added observation errors. During turning, the polynomial model’s positioning accuracy was significantly affected by changes in streamer shape, with the largest deviation occurring around 3750 s. As shown in Figure 4, this moment corresponds to the scenario of maximum curvature. This deviation primarily arose from modeling error, similar to results from the error-free dataset. In contrast, the harmonic model effectively mitigated modeling error, keeping the overall deviation relatively low. After 6000 s, as the streamer shape straightened, the positioning accuracies of both analytical models converged. Additionally, the overall trend matches that shown in Figure 5, with underlying reasons consistent with those discussed in Section 3.1.1.

Table 5 shows the deviation statistics over spatial for the turning scenario. Overall, the polynomial model exhibited a higher mean deviation and standard deviation than the new model, with the maximum deviation also being significantly higher. However, the interquartile range remained the same for both models, indicating similar spatial variation across models. In the straight scenario, both results remained consistent.

**Figure 10:**
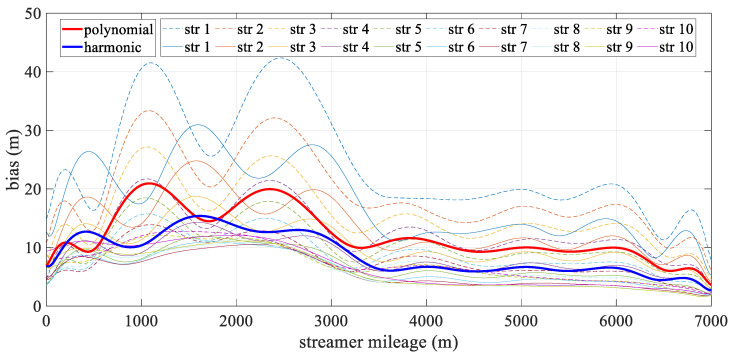
Mean positioning deviation along offset in the turning scenario.

**Figure 11:**
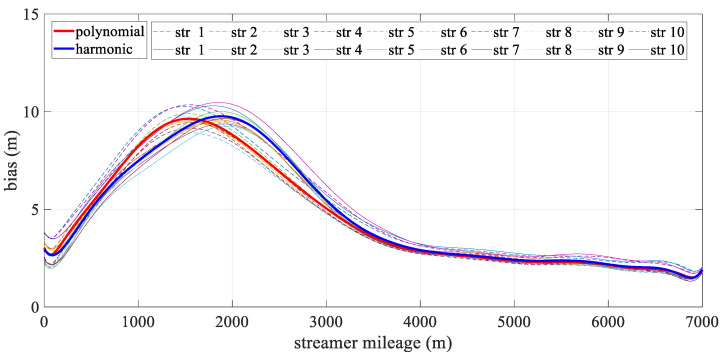
Mean positioning deviation along offset in the straight scenario.

Figure 10 illustrates the spatial mean deviation along the offset during the turning scenario, with the bold line indicating the mean positioning deviation derived from all streamers for both models. The harmonic model showed better performance than the polynomial model. Both models exhibited low positioning errors at the ends of the streamer, with errors gradually increasing toward the front–middle section. This increase was primarily due to the use of front–rear acoustic networks in simulations, where both ends are constrained by acoustic observations and RGNSS buoys. This phenomenon mirrors that shown in Figure 6, as the front–middle section was only constrained by compass data. Figure 11 depicts the mean positioning deviation across offsets for straight scenario, showing that both models achieved even distributed positioning accuracy and similar stability in their results.

**Figure 12:**
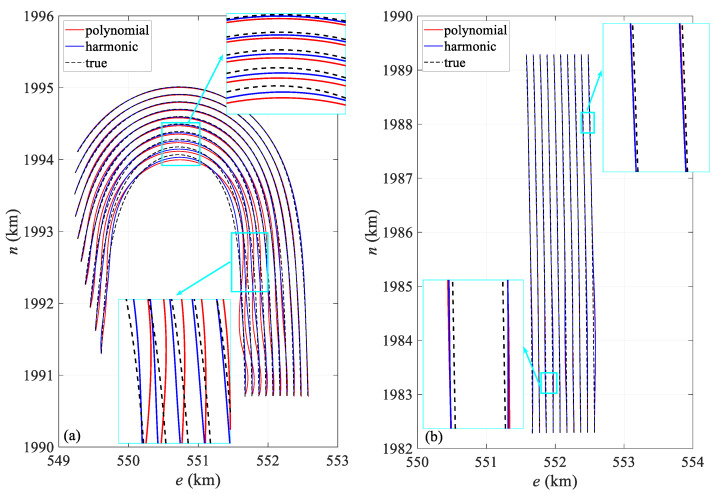
Streamer shape in (**a**) 3790 s turning and (**b**) 8000 s straight scenario.

Figure 12 presents a comparison of the streamer shape in both the turning and straight scenarios. Figure 12a shows a comparison of the shapes calculated from the dataset with an observation error at 3600 s alongside the actual shapes. Similarly to Figure 8a, it captures the moment when the streamer is at maximum curvature during turning. Both models exhibit positional deviations in the calculated shapes, with the errors in the polynomial model primarily arising from modeling inaccuracies. The harmonic model better matches the real shape, but still shows slight deviations in the front–middle section. The modeling error of the polynomial model remains greater than that of the harmonic model, particularly for the innermost streamer. Overall, the harmonic model calculates a more accurate shape. Additionally, there is a noticeable fluctuation between the fitted and actual streamer shapes, mainly due to node distribution, due to similar reasons discussed in Figure 8a. Figure 12b shows the streamer shape at 8000 s, representing the straight scenario, where both models align closely with the actual shape.

**Figure 13:**
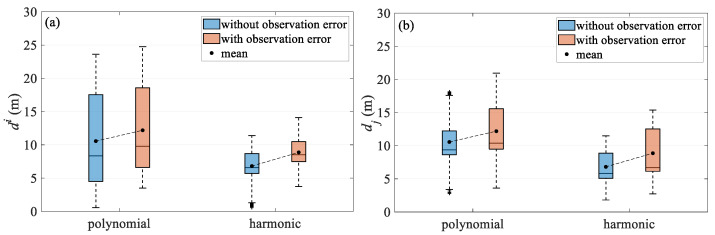
Deviation quartile over (**a**) temporal and (**b**) spatial in turning scenario.

**Figure 14:**
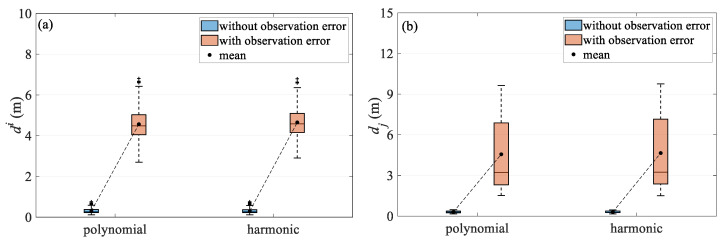
Deviation quartile over (**a**) temporal and (**b**) spatial in straight scenario.

Based on the simulation data test results, the comparison of the quartile plots of the two methods under different observation noise conditions is summarized in Figure 13 and Figure 14, and the test results are further discussed. Figure 13 compares the two models for turning scenario, both before and after the addition of error in the temporal and spatial quartiles. The results show that the harmonic method outperforms the polynomial model under ideal, error-free simulation conditions. When applied to simulation data with errors, the accuracy degradation of both approaches is similar, likely due to their common reliance on parameter estimation using the least squares criterion. In the turning scenario, positioning results are primarily affected by the change in the shape of the towed streamer. However, as the streamer begins to bend, the modeling error of the polynomial model increases rapidly, whereas the proposed harmonic method maintains relatively good performance, suggesting stronger adaptability. This is further confirmed in the simulation test case, where there is added error. Overall, the harmonic function model better reflects the dynamics of the streamer, mitigates observation errors more effectively, and demonstrates greater robustness. Figure 14 compares the two models in the straight scenario, where the positioning accuracy of both models is very close, as the streamer remains relatively straight.

### 3.2. Field Test

Field data collected in January 2024 from a work area in Indonesia were used to test both models, including both turning and straight scenarios. The streamer system was equipped with DGNSS devices, towing a total of 10 streamers, each approximately 8100 m in length. Each streamer’s tail buoy was fitted with an RGNSS, and the gun arrays and tail buoys collectively housed 20 RGNSS devices. Additionally, 30 compasses were mounted on each streamer. The acoustic network consisted of front and rear components: three acoustic transducers were mounted at the head of each streamer, forming a front acoustic network along with transducers on the gun arrays, while 14 acoustic transducers were installed on each streamer in the mid-rear sections to form a rear network. This setup resulted in 648 hydrophones per streamer. Given the relatively stable depth of the streamers in seawater, mean positioning accuracy statistics in this experiment focus solely on horizontal coordinates.

During the field experiments, all positioning sensors were inevitably subject to errors, which caused some inaccuracies in the streamer’s positioning datum. Typically, such errors are considered to be at the decimeter level and are therefore assumed to have little overall impact. However, in addition to random errors, compass and acoustic sensor observations often contain outliers and systematic biases, such as those caused by magnetic field and acoustic propagation errors. In order to obtain reference results for the field data, this study employed professional onboard streamer positioning software, which is able to produce more accurate and towed streamer positioning results. These hydrophone positioning results serve as a comparative reference to assess the accuracy of the proposed method. The difference in hydrophone positioning over epochs and offset are calculated according to Equations (20) and (21), respectively.

#### 3.2.1. Turning Scenarios

**Figure 15:**
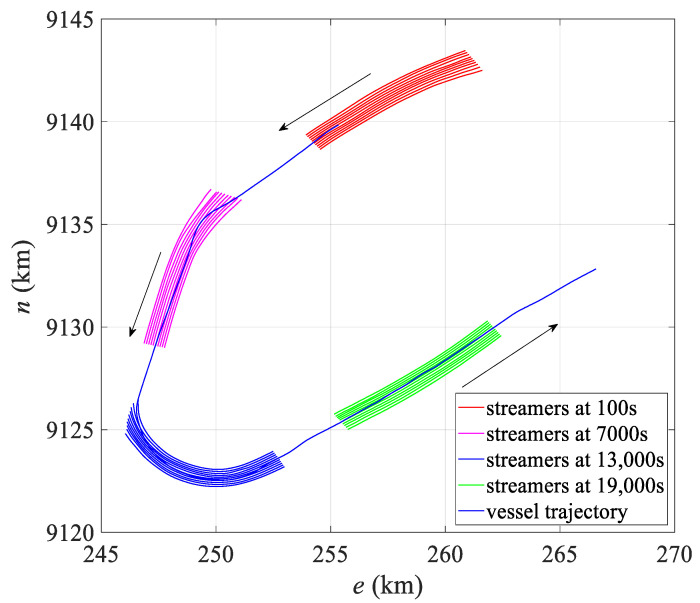
Turning trajectory for field data.

As illustrated in Figure 15, the field data were collected when the streamer executed a 180° turn, with the vessel completing the turn through angles of approximately 140° and 40°, respectively, at a turning radius of about 4000 m, followed by a straight trajectory. This dataset comprises 1066 epochs, with each epoch spaced 20 s apart, totaling 21,320 s.

**Figure 16:**
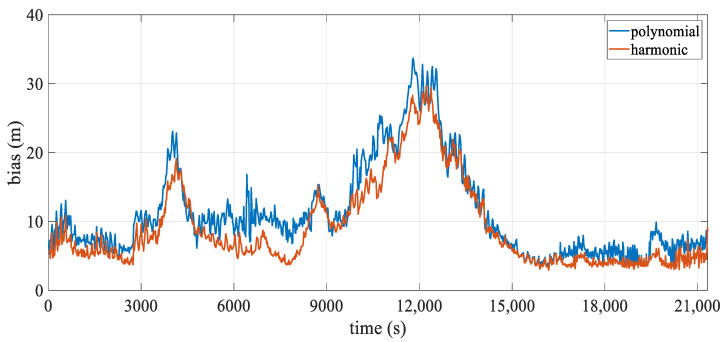
Mean positioning deviation over epoch.

Table 6 presents the statistics of the hydrophone deviation over temporal. The mean positioning deviation for the polynomial model is approximately 11.10 m, while that for the harmonic model is around 9.09 m, resulting in an accuracy improvement of approximately 18.1%. The maximum deviation decreases from 33.69 m to 29.69 m, further enhancing the stability of hydrophone positioning. Figure 16 illustrates the mean positioning deviation over epochs, highlighting two peaks at approximately 4000 s and 12,000 s. As seen in Figure 15, these peaks correspond to moments when the streamer is at the point of maximum turning curvature, with the mean and overall deviation of the harmonic model being lower than those of the polynomial model. Additionally, the deviation around 4000 s is smaller than that around 12,000 s, mainly because the turning radius of the former is larger than that of the latter.

**Figure 17:**
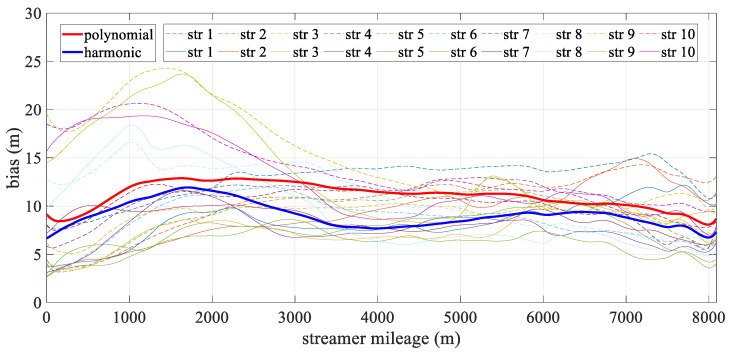
Mean positioning deviation along offset in the turning scenario.

**Figure 18:**
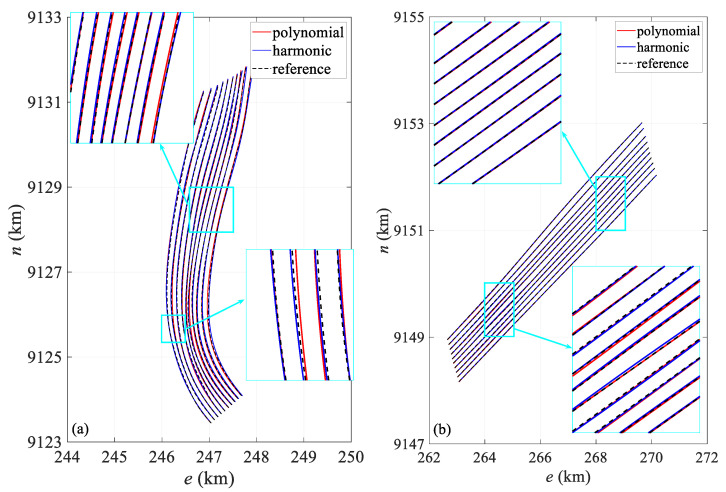
Streamer shape in (**a**) 10,000 s turning and (**b**) 5829.2 s straight scenario.

**Figure 19:**
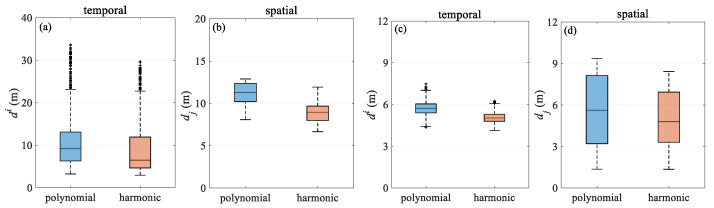
Deviation quartile over temporal and spatial in turning (**a**,**b**) and straight (**c**,**d**) scenarios.

Table 7 provides the deviation of hydrophone spatial statistics for the turning scenario. The harmonic model exhibits a lower deviation compared to the polynomial model, with its interquartile range being notably reduced by 21.8%, suggesting enhanced spatial stability. Figure 17 illustrates the mean deviation along offset, demonstrating that the harmonic model outperforms the polynomial model. Notably, the deviation is primarily concentrated in the front–middle section, particularly for the polynomial model. The primary reason for this is similar to that in Figure 6, where the front–middle section lacks acoustic observations. However, in Figure 17, some individual streamers show large deviations in the front section. Although an acoustic network exists in this section, this is mainly due to the absence of RGNSS head buoys in this section, the lack of the position datum, and the poor quality of the acoustic and compass observations. Figure 18a compares the shapes at 10,000 s in the turning scenario, indicating that the modeling error in the front–middle section of the polynomial model remains larger than that of the harmonic model. Figure 19a,b presents quartile plots of both temporal and spatial models; the mean of the harmonic model is lower than that of the polynomial model, although the quartile spacing is slightly larger, primarily due to moments of larger errors occurring during the two turns.

The field data test results were consistent with the simulation test results, and the deviation of the polynomial model was still larger than that of the harmonic model. The improvement in the field experiment was slightly lower compared to the simulation experiment, possibly due to the difference in turning radius. The field data exhibited a larger turning radius, resulting in a smaller curvature of the streamer. Both models could fit the streamer shape relatively adequately, so there was not a significant difference in the modeling error between the two. Additionally, the bias in the field data can be classified into two categories: modeling error and bias resulting from observation errors and outliers. The reference benchmark data also contained some errors. During the turning experiment, the interaction of multiple errors indicated that the modeling errors were slightly lower and were not the primary source of deviation.

#### 3.2.2. Straight Scenarios

**Figure 20:**
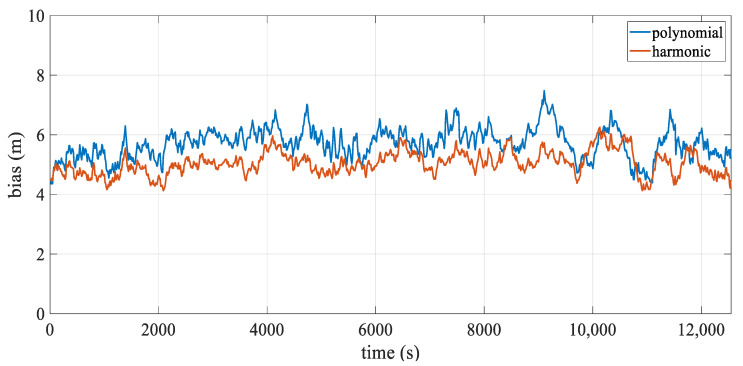
Mean positioning deviation over epoch.

The azimuth of the straight line was 239°, with a time period of approximately 3 h and a total length of about 24 km, including 1076 shot epochs, with an average interval of approximately 11.7 s between shots. Table 8 summarizes the statistics of hydrophone deviation over temporal, showing that both models had a mean positioning deviation of around 6 m, indicating comparable accuracy. Figure 20 depicts the mean deviation across epochs, highlighting the similarities between the two models. However, it also shows that the harmonic model had a lower mean and overall deviation compared to the polynomial model.

**Figure 21:**
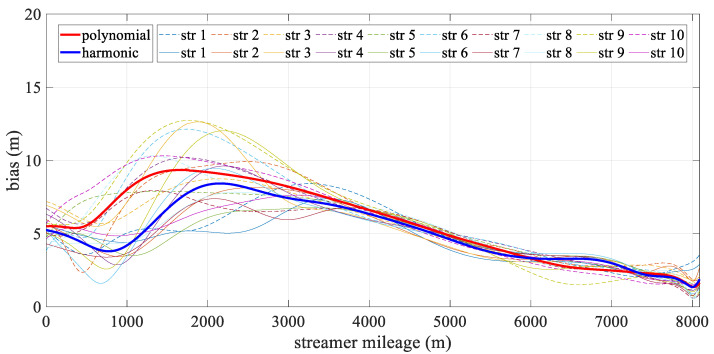
Mean positioning deviation along offset in the straight scenario.

Figure 21 illustrates the mean positioning deviation of hydrophones at different offsets in the straight scenario. The positioning accuracy of both models is uniformly distributed, and the stability of the results is similar. However, larger errors are still primarily concentrated in the front–middle section, which is related to the acoustic network configuration. Figure 18b presents the streamer shape at 5929.2 s, during which the streamer is in a straight scenario, and both models align closely with the reference shape. Figure 19c,d shows the quartile plots of the two models in temporal and spatial for the straight scenario, revealing that the two models are fundamentally similar, although the harmonic model performs slightly better.

In the straight scenario, the accuracy of the two models is the same, but the harmonic function model is slightly more stable than the polynomial model, particularly in the front–middle section. This section is the most challenging area for improving the accuracy of towed streamer models. Therefore, the harmonic function model is better suited for straight scenarios. Additionally, the harmonic function model performs significantly better than the polynomial model in turning scenarios, especially when the streamer experiences large bending, effectively preventing abnormal shapes.

The harmonic models show significant improvements in streamer positioning performance. We see that there are the following several possible factors contributing to this advantage. First, in terms of the number of parameters in the model, neither analytical method reduces solution efficiency as the number of nodes increases. However, compared to the polynomial model, the harmonic model is simpler to fit because it consists only of constant and first-order homogeneous terms, avoiding the oscillations often seen with higher-order terms. Second, streamers are primarily subject to the continuous flow of seawater, including currents and tides such as ocean currents, littoral currents, and tidal currents. The marine environment exhibits dynamic fluctuating properties, and the harmonic function provides a better representation of these dynamics. Finally, the harmonic function term allows the actual length of the streamer to be set and ensures that the streamer length matches its actual physical length, rather than treating the offset along the streamer merely as an independent variable. This alignment is essential for accurately controlling the reconstructed streamer length.

## 4. Conclusions

In marine towed-streamer seismic exploration, accurate hydrophone positioning is crucial for acquiring high-quality seismic data, and high accuracy, stability, and applicability are the primary performance criteria for streamer models. Although existing streamer models effectively describe the streamer shape in a straight scenario, the analytical polynomial model exhibits large modeling errors during large curvature turns. To address this issue, this paper proposes a novel multi-streamer analytical positioning method that employs hybrid harmonic functions for modeling. The error equations and process are derived in detail and validated using both simulation and field data. The experimental results indicate that the hybrid harmonic model significantly enhances hydrophone accuracy in turning scenarios compared to the analytical polynomial model. It effectively mitigates the model errors associated with large curvature conditions, while the performances of both models are comparable in straight scenarios. The two models share similar solution parameters, but the new model is less complex. The hybrid harmonic function terms consist solely of homogeneous term components without higher-order terms. This simplification avoids the overfitting issues commonly associated with high-order terms and notably improves the stability of hydrophone positioning results. Furthermore, the inclusion of the nominal length of the streamer within the harmonic function terms helps to reduce oscillations that can arise at the edges of intervals when using high-order polynomial fitting. It also addresses model bias resulting from poor-quality observations at the edges of the streamer, thereby enhancing model stability. However, the periodic terms within the harmonic function require further investigation in order to identify and refine their specific periodic components. Additionally, the order of the harmonic function remains empirically determined, and its influence on the relevance of the model needs further exploration. Moreover, integrating harmonic functions with tidal or current models presents a promising avenue for enhancing the accuracy of the streamer model. Furthermore, this approach offers a new perspective for streamer model construction and error handling, advocating for a departure from single-model reliance toward the integration of multiple functional characteristics. Using diverse types of functions for modeling can better address complex scenarios. How to further simplify and accurately model the streamer is still an important research direction to address underwater complex scenes more effectively. Meanwhile, the hybrid harmonic functions can not only serve as the basis for curve integration, which helps to develop more rigorous models for improved computational efficiency, but can also be used for coordinate integration during data preprocessing, resulting in highly stable approximations of nodal coordinates. Similarly to cubic splines, segmenting harmonic functions can further minimize modeling errors, leveraging the smoothness constraints at the joints to align more closely with the physical properties of smooth curves.

## Figures and Tables

**Table 1:** Configuration parameters for simulation.

Configuration Type	Parameter Name	Value
system configuration	streamer separation	100 m
	gun array separation	50 m
	magnetic declination	−1.88°
	survey line azimuth	0°
sailing configuration	vessel speed	2.0 m/s
	seawater density	1000 kg/m^3^
	speed of sound	1500 m/s
	northward current	0 m/s
	eastern current	0 m/s
streamer physical properties	streamer diameter	0.0737 m
	unit mass	4.266 kg/m
	young’s modulus	1.0314 × 10^12^ N/m
	tangential drag coefficient	0.0025
	normal drag coefficient	1.7

**Table 2:** Statistics of positioning deviation over temporal (unit: m).

Scenario	Model	Mean	Standard Deviation	Max	Min	Interquartile Range
Turn	Polynomial	10.56	7.27	23.60	0.57	13.05
	Harmonic	6.81	2.66	11.38	0.57	2.97
Straight	Polynomial	0.30	0.11	0.76	0.12	0.15
	Harmonic	0.30	0.12	0.76	0.12	0.14

**Table 3:** Statistics of positioning deviation over spatial (unit: m).

Scenario	Model	Mean	Standard Deviation	Max	Min	Interquartile Range
Turn	Polynomial	10.56	3.40	18.14	2.79	3.60
	Harmonic	6.81	2.32	11.50	1.81	3.81
Straight	Polynomial	0.30	0.09	0.48	0.15	0.13
	Harmonic	0.30	0.08	0.45	0.15	0.13

**Table 4:** Statistics of positioning deviation over temporal (unit: m).

Scenario	Model	Mean	Standard Deviation	Max	Min	Interquartile Range
Turn	Polynomial	12.19	6.68	24.76	3.50	11.95
	Harmonic	8.87	2.37	14.09	3.73	3.00
Straight	Polynomial	4.56	0.72	6.80	2.70	0.97
	Harmonic	4.65	0.70	6.79	2.90	0.94

**Table 5:** Statistics of positioning deviation over spatial (unit: m).

Scenario	Model	Mean	Standard Deviation	Max	Min	Interquartile Range
Turn	Polynomial	12.19	4.27	20.94	3.61	6.08
	Harmonic	8.87	3.46	15.38	2.73	6.39
Straight	Polynomial	4.56	2.67	9.63	1.51	4.58
	Harmonic	4.65	2.73	9.76	1.49	4.78

**Table 6:** Statistics of positioning deviation over temporal (unit: m).

	Mean	Standard Deviation	Max	Min	Interquartile Range
polynomial	11.10	6.67	33.69	3.24	6.81
harmonic	9.09	6.21	29.69	2.94	7.28

**Table 7:** Statistics of positioning deviation over spatial (unit: m).

	Mean	Standard Deviation	Max	Min	Interquartile Range
polynomial	11.10	1.33	12.89	8.08	2.16
harmonic	9.09	1.30	11.93	6.66	1.69

**Table 8:** Statistics of positioning deviation over temporal (unit: m).

	Mean	Standard Deviation	Max	Min	Interquartile Range
polynomial	5.71	0.50	7.48	4.36	0.65
harmonic	5.05	0.39	6.25	4.13	0.51

## Data Availability

The authors do not have permission to share data.
